# Behavioral Patterns in Breaking Bad News Communication: An Ethnographic Study with Hematologists

**DOI:** 10.3390/ijerph19052585

**Published:** 2022-02-23

**Authors:** Giovanna Artioli, Luca Ghirotto, Sara Alquati, Silvia Tanzi

**Affiliations:** 1 Palliative Care Unit, Azienda USL-IRCCS di Reggio Emilia, 42122 Reggio Emilia, Italy; sara.alquati@ausl.re.it (S.A.); silvia.tanzi@ausl.re.it (S.T.); 2 Qualitative Research Unit, Azienda USL-IRCCS di Reggio Emilia, 42122 Reggio Emilia, Italy; luca.ghirotto@ausl.re.it

**Keywords:** bad news communication, palliative care, training, ethnography

## Abstract

Hematologists adequately disclosing bad news is a critical point precluding patient-centered communication. Specific courses on communication for hematologists seem to be rare, as well as research exploring their communicative skills and patterns. We aim at describing the hematologists’ behavior during difficult conversations to account for behavioral patterns in communication and provide new insights regarding teaching skills to communicate bad news. We employed a focused visual ethnography to answer the following research: “what are hematologists’ behavioral patterns in communicating bad news to patients and families?” The collected data included (1) video recordings, (2) observational field notes, (3) interviews with hematologists. The analysis highlighted four patterns: (1) a technical-defensive pattern, (2) an authoritative pattern, (3) a relational-recursive pattern, and (4) a compassionate sharing pattern. Hematologists seem to have difficulty expressing compassionate caring and empathetic comprehension. Communication skills remain a challenge for hematologists. The study of behavioral patterns can lead to increasingly targeted training interventions for this specific learner population.

## 1. Introduction

Patients with hematological malignancies are unique in terms of the challenges they face during their illness until the end of their life, as the trajectory of the disease is irregular and unpredictable [1,2,3]. In addition, the severity of a hematological malignancy diagnosis also heavily depends on the medical team involved, who typically develops close and long-term relationships with their patients [4]. Therefore, the right conditions (timing and the doctor-patient relationship) to share bad news may not be clear. The bad news is defined as any news that harms individuals’ future perceptions [5,6]. Communicating bad news includes communicating an adverse diagnosis and prognosis and communication about the disease’s progression from cure-focused to comfort-focused health care [7].

In hematology, the literature related to communicating bad news is still scarce even though the difficulties hematologists have in recognizing the poor prognosis of patients and disclosing that information to them are widely reported [1,8,9]. Discrepancies between the hematologists’ communication of the prognosis and the patients’ understanding have also been highlighted [10]. Several factors limiting patient-centered communication in the hematology context have been investigated: insufficient information exchange, the misalignment of treatment goals, and discordant role preferences in treatment decision making [11].

Consequently, learning and practicing good communication skills is compelling; skills to practice mainly include sharing bad news and helping patients and families negotiate difficult decisions [12,13]. In addition, hematologists have acknowledged that they take a paternalistic approach towards choices and explain their therapeutic optimism to support patients during toxic but curative treatments [14,15]. It has been outlined that hematologists may overtreat patients due to intense doctor-patient relationships in the setting of a treatments’ unpredictable nature. Furthermore, the therapeutic possibilities allow hematologists to think, “Maybe we can pull another ‘rabbit out of the hat’” given the options offered by different therapeutic lines [16]. Honest communication and transparent treatment proposals to share with patients and their caregivers are strongly intertwined variables [17,18].

Some researchers have called for formal training in communication skills and targeted interventions for patients with hematologic malignancies from palliative care staff [4,19]. While in oncology [20,21] and palliative care [22,23], studies have been conducted on training healthcare professionals concerning the communication of bad news—including the implementation of protocols and guidelines to make such communication more efficacious [24,25,26]–to the best of our knowledge, evidence concerning hematologists’ skills, communicative behaviors and educative needs in this context is scarce.

Since there is room for improvement in communication in hematology, in 2015, a group of psycho-oncologists and palliative care specialists, working in a Palliative Care Unit within a general hospital, implemented training on communicating bad news, which was described in a recent publication [27] (the Teach-to-Talk program). The training was designed by adopting different teaching methods: lectures, role-play, bedside training. A behavioral checklist was used for tutoring trainers to evaluate communication skills [28].

In the context of this training, we aimed at describing the hematologists’ behavior during difficult conversations to account for behavioral patterns in communication and provide new insights regarding teaching skills to communicate bad news. We so explored the behavior of hematologists under challenging conversations during both this specific training and in real-life situations, applying an ethnographic approach. No similar study has been conducted so far with hematologists. Specific objectives were: (i) analyzing in-depth the behaviors of hematologists during their role-plays; (ii) observing the behaviors of hematologists during complex communications in clinical practice; (iii) gathering the perceptions of hematologists in this regard. Accordingly, we formulated the following research question: “What are the hematologists’ behavioral patterns related to communicating bad news to patients and families?”.

## 2. Materials and Methods

We applied an ethnographic approach previously used by Ng and colleagues for studying health professionals’ education and practice [29]. Using this approach, we employed a specific method called focused ethnography [30]; it entails entering the field with a defined research question [29], undertaking fieldwork in a short timeline [31], and restricting the field of inquiry to a specific phenomenon within a planned event with key-informants [32]. Consequently, its results do not entail lengthy records but instead produce rather direct and short reports, providing insights into the narrow research focus [31]. Visual data is integral in this focused ethnography [33], connecting it with visual ethnographic traditions. For our study, we formulated the following research question: “What are the hematologists’ behavioral patterns related to communicating bad news to patients and families?”. The study report follows the SRQR checklist [34], which is provided Supplementary File S1.

### 2.1. The Organizational Setting

The present work was carried out at the Hematology Department of a Comprehensive Cancer Centre in Northern Italy. Here is a Palliative Care Unit whose goals are to develop and implement training programs to improve health professionals’ palliative care competencies, especially communication. This unit is a specialized hospital-based unit with no beds. At present, it includes three senior palliative care specialists (physicians), and two advanced practice nurses, with a remit of specialist consultations in different wards and a clinic for advanced outpatients and their relatives.

The Hematology Department provides inpatient and outpatient services. The medical staff includes up to 15 hematologists organized according to their different areas of expertise.

### 2.2. The Training

A training program titled the “Teach-to-talk” was implemented within the participants’ hospital ward [27]. The trainers were professionals from the Palliative Care Unit. Different teaching methods were employed: lectures, video-recorded role-playing, briefing sessions, and bedside sessions during real patient encounters.

Role-playing sessions were organized involving no more than four hematologists each. Two were asked to act as role-play characters while the others were observers. Two consecutive role-playing sessions were scheduled, lasting about one and half hours. The participants proposed the communication scenarios based on real situations they experienced in the field. The bad news to communicate entailed the following topics: the end of active treatments, poor prognosis, and interference from relatives/caregivers [27]. Roleplaying scenarios and structures are shown in Table 1.

To conduct the briefing sessions, we referred to the method proposed by Rudolph and colleagues [35], entailing three co-participated activities, i.e., reaction, analysis, and summary.

The bedside sessions were assessed using the Breaking bad news Assessment Schedule (BAS), a structured method (checklist) for rating professional skills at breaking bad news, especially in evaluating videotapes or observing actual consultations [28]. Furthermore, BAS provides information on the different components of the breaking bad news consultation (setting the scene; breaking the news; eliciting concerns; information giving; and general considerations) [28].BAS was translated but not validated into Italian. Still, it did fit the training’s goal since it contains specific and relevant items for good observation of communication exchange. During the bedside session, trainers filled the BAS, and afterward, the resulting scores were shared and discussed with trainees.

All the hospital’s hematologists participated after the head of the department called for training.

### 2.3. Sampling and Participants’Recruitment

Participation in the training implied involvement in the study. The participants comprised all the hematologists (n. 15) working at the Hematology Department.

### 2.4. Data Collection

The data collected regarded the trainees as they were attending the course. We could collect several types of data:

- Video recordings of role-playing sessions.
- Observational field notes (taken during bedside sessions along with filled BAS checklists [28], whose results were shared with trainees).
- Semi-structured interviews, conducted with hematologists after the end of the training.

Those three separate processes of data collection allowed us to explore participants’ behaviors in three different conditions: during fictional situations (role-playing sessions) to be compared with external observations of real-life bad news communication events (also informed by the BAS’ scores), and participants’ first-person narratives, to have rich data explaining also real contexts’ communicative patterns.

The principal investigator, GAwas involved as an observer in 14 role-playing sessions, video recorded. The recordings were then descriptively transcribed. GA and LG put the transcriptions into a table and annotated the patients’ nonverbal behaviors during role-playing next to the participants’ words, along with providing observational notes.

As the role-playing sessions were finished, the sessions were conducted at the patient’s bedside observations were made. Immediately after the bedside, the training facilitators documented observational field notes obtained from the bedside by ST and SA.

One year after the end of the training, GA administered semi-structured interviews with nine hematologists to explore three main topics:

- Their experiences with difficult communication during their clinical activity.
- Their perceived emotions and feelings.
- The content of their difficult communication.

The interviews allowed the researchers to contextualize, in clinical practice, what has been identified in the observation of simulated situations. The interviews were audio-recorded, transcribed verbatim, and anonymized. Transcripts were not returned to the participants.

Taken together, the data collected through different collection strategies and within a variety of situations allowed researchers to reach what hematologists had to say about why they behaved as they did [36].

### 2.5. Data Analysis

GA began the analysis while collecting data, as Roper and Shapira indicate [37], and the data was then triangulated [38].

The first analysis concerned the role-playing sessions. Participants’ verbal and nonverbal communication behaviors and observational notes were labeled and described to facilitate further analysis.

Then, bedside sessions’ fieldnotes and interview transcripts were subsequently added to the data set. No statistical analysis was done upon the BAS scores. The scale was not validated in Italian, the number of participants was low, and the trainers used it mainly to inform field notes on the participants’ behavior.

The whole data set was analyzed following a five-step process [39], sustaining the comparison of emerging patterns [37] across the different kinds of data (triangulation):

1. Sorting of collected material (all the data were read extensively).
2. Descriptive coding of observational data and interviews (GA descriptively labeled the data, with codes’ names close to the raw data. The researcher reviewed all data line by line and identified words, phrases, and events). Preliminary interpretative findings were also defined [40].
3. Questioning data to find similarities and differences (GA organized codes and formatted them into tables according to data type. Then, she wrote descriptive accounts of the data from the different sources. The authors reached an agreement about the main similarities and differences emerging during this step).
4. Grouping codification labels into behavioral patterns and challenging first interpretations (GA and LG grouped codes into categories. The emerging behavioral patterns were compared with raw data and descriptive accounts to generate new insights into the data [40]).
5. Comparing participants’ meanings and categories and defining final interpretations [32,39] (authors added meaningful quotations and field notes excerpts to the final report).

### 2.6. Rigor and Reflexivity

The data collection was carried out iteratively. Since this study was a focused ethnography and required descriptions of a particular event, saturation could not be applied [41]. Nonetheless, rigor within the analysis was assured by using qualitative trustworthiness criteria [42]. To address credibility, the analysis process was scrutinized by all research team members. Confirmability is demonstrated by presenting examples of the data (Table 2) and reducing the risks of individual researcher bias since all the team members were involved in the analysis process. Different data sources and collection provided a “thick description” of the hematologists’ behaviors. About transferability, the findings of this study provide specific patterns, which other researchers can use to compare, contrast, or expand upon during their research in different cultures and settings.

As to reflexivity, GA, the external researcher in this study, has a master’s degree in nursing and works as a research nurse in the Palliative Care Unit. GA is an RN, MSc in Nursing with specific expertise in communication (she holds a MA in communication for health professionals). She is also working at the university level as a lecturer in a communication laboratory. She had previous experience conducting qualitative studies as a tutor for the master’s degree in Palliative Care. As to the other researchers, SA (oncologist and palliative care specialist) and ST (Ph.D. in Experimental Medicine, oncologist, and palliative care specialist) are trainers in palliative care communication. They supervised the training. All had prior experience developing and leading communication courses in oncology and palliative care [27,43,44]. ST and SA had frequent contact with hematologists as consultants in palliative care. LG, a background in education and social science research and an expert in qualitative methodology, served as a” critical friend” during the whole research process.

## 3. Results

The hematologists were ten males and five females. Their average age was 46 years old (range 36–60), and their average work experience was 16 years (range 4–32). Fourteen role-playing scenarios with different themes were video recorded, as shown in Table 1. Fourteen bedside sessions were performed, ultimately involving 11 out of the 15 hematologists.

Analyzing the data collected throughout the ethnography highlighted four cross-cutting categories: (1) a technical-defensive pattern, (2) an authoritative pattern, (3) a relational-recursive pattern, (4) a compassionate sharing pattern.

1. The technical-defensive pattern emerged whenever hematologists employed technical and clinical terms, which were difficult for the patients and caregivers to understand.
2. The authoritative pattern emerged whenever doctors employed their authoritativeness to explain the situation to the patients and the caregivers.
3. In this pattern, the relational-recursive way allows the doctors to invest in a relationship with the interlocutors to convey the chances for recovery. Hematologists restored the patients’ history using narratives marked by positive thinking and sharing opportunities for care.
4. Compassionate sharing pattern: in this pattern, a human relationship could be established by doctors whenever they activated reciprocity and sharing in the conversation, with particular attention to patients’ moods and emotions.

While BAS scores from the bedside sessions are shown in the Supplementary File S2 to account for where the bedside field notes were derived and compared, Table 2 summarizes the behavioral patterns, related data, and observational notes regarding language the participants used.

### 3.1. Technical-Defensive Pattern

This pattern included doctors being the main ones speaking, leaving little space for interlocutors. In this context, patients/caregivers could not have the chance of posing questions or confirming their understanding.

In addition, bedside evaluations and observations confirmed this pattern throughout all the encounters. The facilitators highlighted technical terms and unrequested information concerning the disease in the BAS and the observational notes.

Under the technical-defensive pattern, we could recognize the following three subcategories: using clinical content, not mentioning the pathology, using a pressing tone. This pattern, along with subcategories and meaningful quotations from participants or data, is summarized in Table 2.

#### 3.1.1. Using Clinical Content

This subcategory described when hematologists focused on clinical content, not very useful or not understood by the interlocutor.

As to item 6 in the BAS, 5 out of 6 participants used technical terms during bedside sessions and did not adjust their language to the patients’ level. In this context, the facilitator observed embarrassment, first, and then the hematologists calmed down as the conversation progressed, eventually giving information that patients had not requested.

Behaviors we observed included: difficulties in maintaining eye contact, a static body posture and facial expression, a regular and flat voice tone.

#### 3.1.2. Not Mentioning the Pathology

Hematologists avoided directly naming the disease, replacing it with paraphrasing. This subcategory emerged mainly from role-playingsessions’ analysis. A doctor tried to alleviate the bad information during a role-playing session by using weaker words.

#### 3.1.3. Using a Pressing Tone

Finally, a defensive approach occurred when the doctors used a high-pitched tone that overpowered the patients/caregivers’ voice, even when they admitted their difficulty giving pertinent answers.

### 3.2. Authoritative Pattern

The authoritative pattern entailed verbal, paraverbal, and non-verbal observations, which showed a high-pitched tone of voice and a prevalence of firm posture and still facial expressions. Despite the bad news, the doctors pointed out that there were still several possibilities for a cure on a scientific basis. Clinical skills helped the doctor communicate.

In addition, this pattern was confirmed in the bedside observational notes and the BAS score for item 21.

This pattern was based on two main behaviors: hematologists use their expertise and convince the patient and their family of the possibilities.

#### 3.2.1. Using Their Expertise

The doctor used his expertise to provide patients and family members with opinions based on their authority and clinical experience.

#### 3.2.2. Trying to Convince

An authoritative pattern also emerged when doctors tried to ‘convince’ the patients about what were, in their opinion, the best choices for treatment and quality of life.

Additionally, this aspect emerged in BAS item 14, which was focused on this behavior, and all hematologists demonstrated it from time to time.

### 3.3. Relational-Recursive Pattern

This behavioral style emerged when a doctor-patient relationship was established. This pattern included an appropriate alternation between talk and silence. The tone of the voice appeared modulated and adapted to doctors’ communication contents. Postures, gestures, and facial expressions were more dynamic and alternated during the conversations from role-playing sessions. During bedside sessions, thirteen hematologists used this pattern, as confirmed in BAS items 7 and 11.

Three essential features characterized the recursive-relational pattern: listening to the person, paying attention to the persons’ emotions, using a narrative approach.

#### 3.3.1. Listening to the Person

This pattern emerged whenever doctors demonstrated to listen to the interlocutor actively. Here, doctors were inclined to put the interlocutors at ease.

During the role-playing sessions, those who acted as patients/caregivers felt comfortable to reply to doctors.

#### 3.3.2. Paying Attention to the Persons’ Emotions

Connected to the previous subcategory, doctors could also be attentive to the persons’ doubts’, welcoming their feelings.

Paying attention to feelings also appeared to give hope in honest communication.

#### 3.3.3. Using a Narrative Approach

Some hematologists applied an information-giving approach within a narrative frame, accounting for the patients’ disease history. It is worth mentioning that the narrative approach was taught during the training program and in each case applied, especially during real encounters with the patients at their bedside.

Hematologists could take advantage of their long-lasting relationship with a long-standing knowledge: accordingly, items 2 and 4 in the BAS scored high.

### 3.4. Compassionate Sharing Pattern

Once self-awareness with a conscious use of silence and the hematologists developed active listening, a compassionate sharing pattern arose. It entailed a prevalence of dynamism and body movements (i.e., leaning towards the person, modulated body language, adapting voice tone to the understanding needs of the interlocutors).

The compassionate sharing pattern consisted of three subcategories: paying attention to the setting and controlling emotions; applying compassion and empathy; sharing care.

#### 3.4.1. Paying Attention to the Setting and the Control of Emotions

The participants could create a ‘favorable’ climate when an effective relationship was established. In this subcategory, the hematologists recognized that attention to the setting was pivotal.

This subcategory was confirmed for all hematologists in BAS item 1. Twelve hematologists appeared supportive during the real encounters (items 19 and 20).

#### 3.4.2. Applying Compassion and Empathy

This subcategory emerged when doctors could show sensitivity.

Compassion and empathy emerged when doctors could wisely give a pace to communication.

Nonetheless, this pattern was critical for hematologists during bedside sessions. Their analysis reported that 29% of hematologists did not exhibit sensitivity when sharing bad news (BAS item 5), and 5 out of 14 hematologists did not explore the patients’ concerns (item 10). Moreover, 5 out of 14 hematologists mentioned supposed patient concerns in item 17. In an observational note, we wrote: “the learner ‘sticks’ his worries on the patient.” Moreover, 9 out of 14 hematologists did not explicitly explore which quality-of-life dimensions the patients found important (item 11).

#### 3.4.3. Sharing the Care

This subcategory described how communication with patients and caregivers was reached. It emerged when doctors agreed to the care.

The analysis of the bedside sessions confirmed this hematologist’s ability to end the encounter at the right moment. Only a participant did not correctly manage timing (BAS item 23).

## 4. Discussion

This study explored the hematologists’ behaviors in communicating bad news providing a contextual-specific interpretation of communicative patterns. Four behavioral patterns were identified: a technical-defensive pattern, an authoritative pattern, a relational recursive pattern, and a compassionate sharing pattern. These patterns were widely observed among the group of hematologists and derived from both the intrinsic characteristics of the group and the training they attended. The significant difference between the simulation data (the interviews and the role-playing sessions) and the real-life context of the bedside sessions is related to the compassionate sharing pattern; the palliative care physicians observed that the hematologists had difficulty expressing compassionate caring and empathetic comprehension. These two essential communication skills were only utilized by some hematologists, even though they had been taught by the palliative care physicians and were recognized as crucial in difficult conversations.

Some hematologists didn’t explore the quality-of-life dimensions, which were not strictly linked to the disease; the hematologists were excellent specialists in their discipline but were not as competent in the patient’s life, which is an attribute more typically associated with palliative care physicians. The scarce literature confirmed these data on this topic [8,9,12]; it has shown a tendency among hematologists to ‘broadcast’ using monologues, mainly while delivering bad news during hematological cancer consultations.

In Alexander and colleagues [8], audio-recorded consultations between patients and hematologists were analyzed; the hematologists commonly discussed the disease and treatment but less widely discussed other, more patient-centered issues such as decision-making preferences and information preferences, similar results in our study As specialized professionals in PC and facilitators of communication training [27], we try to teach serious attention to the values, preferences, quality-of-life dimensions, and concerns of our patients during difficult conversations; this type of active listening and attention is fundamental for being supportive and being helpful to vulnerable patients in the advanced stages of a disease. As shown in the authoritative pattern, Hematologists frequently provide rich recommendations during their discussions about treatment. Still, on the other hand, when no treatment option is the case, recommendations are succinct. Additionally, in the study by Alexander and colleagues [8], quality of life and the impact of treatment were under-evaluated; we believe that the hematologists felt confident and competent with topics such as disease treatment and tried to avoid other complex issues, regardless of patients’ wishes to discuss them.

Unlike the study by Alexander et al., our ethnographic research was enriched by analyzing nonverbal behaviors. Especially in the compassionate sharing pattern, the analysis allowed us to underline the importance of professional compassion, a communication behavior recommended in our training course that could be taught and practiced [43].

In the study by Chhabra and colleagues [9]—which started with the same training as in the study by Alexander et al. [8], the HEMA-Comm—the authors analyzed 20 consultation visits to study the behavioral patterns that emerged among hematologists; the authors found four main patterns (broadcasting, deferential, directive and inviting). The most ubiquitous finding was a pattern of lengthy physician monologues on disease mechanisms and history, treatment options, or prognostic information. There was no room for exchange during the encounters, and the doctors conveyed only information about what was suitable for the patients. In this approach, the patient’s values are not explored, choices are not made according to the patient’s preferences.

Authoritative and technical-defensive patterns may help hematologists establish credibility and provide a general overview of relevant information important objectives of these visits. Still, the concerns and needs of patients could be different. In palliative care, treatments involving chemo- or immunotherapy are not the only answer; therefore, hearing the desires and preferences of the patients opens new dialogues and possibilities. Relational recursive behaviors show active listening and knowledge of patient history and explore patients’ disease awareness. This pattern was a focus in the training course.

As identified in a recent review [45], the greatest challenge in simulation-based qualitative research is the uncertain generalizability of simulations to reality. However, the triangulation of data, as shown here, can improve the reliability of qualitative findings, and a research design is the most effective when it involves experience-based experts, that is, people with lived experiences of the phenomena being explored, as was the case for our population [46].

## 5. Conclusions

The study of patterns can lead to increasingly targeted training interventions for specific learner populations to evaluate the patient’s values and preferences. In this way, communication training should teach advanced communication skills, which does not involve hematologists just convincing patients of their specialist point of view but truly sharing care choices with the patients. As for hematologists, avoiding standard behavior patterns would foster personalized communication with patients without feeling uncertain about complex issues.

Our study suggests a potential future training program on difficult communication should teach attention to all dimensions of patients’ lives as a whole. Active listening to real patient concerns is a challenge for hematology specialists. It is possible that broader training, including more PC competencies, should promote this advanced communication skill.

## Figures and Tables

**Table 1:** Roleplaying structures.

Roleplaying Number	Interview Setting	Hematologist’s Suggestions	Complicating Factors
Purpose: Communication of the Disease Progression			
1	Communication with a woman with Hodgkin’s recurrence	A compassionate drug is proposed	The woman does not seem to have understood her illness well; she has severe anxiety
5	Interview with a patient in an advanced stage of the disease	The patient is expected to change therapies	The doctor has a hard time creating an empathetic relationship with the patient and his wife.
6	Interview with a patient who has already undergone a bone marrow transplant with no success	Palliative therapy is proposed after the other treatments stop being effective	The patient has already undergone several lines of treatment with no results
8	Interview with a patient whose disease is progressing	It is proposed that chemotherapy be resumed	The patient had already stopped therapies in the past, only to resume them then and then have to stop again due to the disease’s progression
13	Interview with a patient with leukemia	The doctor seeks to understand how the patient will manage his life as the disease worsens	There is a daughter in the family with disabilities.
14	Interview with a patient with hematological pathology	The patient is in disease progression, and hospitalization is proposed	The patient is depressed and does not want to accept hospitalization, as treatment no longer makes sense to him
Purpose: Communication of a Poor Prognosis			
10	Interview with a patient with hematological disease	Communication of the poor prognosis directly to the patient	Complex discussion in which the doctor must talk to the patient about his situation, explaining the terminal phase his pathology is entering
11	Interview with a patient with a new oncological pathology	Communication of the prognosis of a new disease and its treatment	The doctor has to talk about diagnosis and a new therapy to a patient who has already been treated with chemotherapy for Hodgkin’s lymphoma
Purpose: Proposing a New Treatment			
7	Interview with a patient with leukemia	Monoclonal therapy is proposed in anticipation of transplantation	The patient is very anxious
12	Interview with a patient diagnosed with myeloma	The doctor proposes chemotherapy	The patient refuses any type of therapy for fear of side effects
Purpose: Communication of the Disease Progression			
2	Communication with the patient’s caregiver (daughter)	It is proposed that the patient move from active care to palliative care due to the disease’s progression and the refractory disease	The daughter does not want the communication to be shared with her mother and does not accept the proposal of hospice
3	Communication with the patient’s caregiver (wife)	The patient has experienced a sudden and severe deterioration and needs support	The caregiver is shocked by the sudden and worsening evolution of the patient’s condition. In addition, the doctor and patient know each other
4	Communication with the patient’s caregiver (wife)	The patient has experienced a sudden and severe deterioration and needs support	The caregiver pours all her anger onto the doctor and tries to attack him.
Purpose: Communication of a Poor Prognosis			
9	Communication with the patient’s wife	Communication of the poor prognosis	The patient is the father of 4 children, and his wife is in a precarious economic condition

**Table 2:** Behavioral patterns, data, and specific language-related features.

Focused on…	Subcategories	Data	Verbal Language	Paraverbal Language	Nonverbal Language
Defensive-Technical Pattern					
… clinical content as a defense mechanism	(a) used clinical content	The doctor seems to have had difficulty since the beginning of the interview, and he/she shows it by not maintaining eye contact, and his/her movements highlight this. He/she looks nervous and uncomfortable with the questions (ON-RP 9) The doctor appears closed off from the patient’s problems and shows a lack of attention” (ON-RP6) “[There was] too much information… she started all in one go but then she asked for confirmation and was unconfirmed… bad time management” (FN) Doctor: “Moreover, platelets were the first problem (…) then from the TC scan we see fungal pneumonia, by aspergillus … and oxygen is no longer enough to secure respiratory exchanges” (RP3)	Prevalent use of clinical content	The tone of the voice is always the same, flat, sometimes pressing	Static posture and facial expressions
	(b) did not mention the pathology	Doctor: “We can’t know for certain … but it can be something worse than what you had previously” (RP2)			
	(c) used a pressing tone	Doctor: [With a loud and determined voice]: “I have an offer for you. We will certainly not do the last type of treatment”. (RP 6) ‘Caregivers’ questions gave the doctor trouble; these are not topics he deals with, and he admits embarrassment about speaking of this because he can’t give a proper answer.’ (ON-RP 9)			
Authoritative Pattern					
… convincing the patient/caregiver	(a) using own expertise	Doctor: “Ok! … But if we set up a therapy that serves to control certain symptoms and … certain pains, we must, however, follow this therapy. … We cannot take it partway.” (RP 2) Doctor: “Well, I say that this pain treatment, to me, is not good … The CT scan didn’t give us good news, and the treatments didn’t give us the expected results.” (RP8) ‘He tried to give an image of himself as having the situation under control’ (FN 5)	Words still prevail over other forms of communication	Sometimes moderate, sometimes used a high-pitched tone of voice	Prevailingly static posture and facial expressions
	(b) trying to convince the patient of a specific treatment option	Patient: “And if ‘the beast’ should reappear”? Doctor: “We will find another treatment to use, but we have to pursue one goal per day; I understand that with this mindset, moving on is hard.” Patient: “Yes, I go on… I always go on…” (RP7) Patient: “There’s no point in continuing the treatment.” Doctor: “The disease is continuing. This treatment can hold the illness, and you can go on living for months with a good quality of life.” (RP 6) Physicians tend to underscore positive outcomes to reduce patients’ expression of bad emotions or sugarcoat the pill. (FN 2-3)			
Relational-Recursive Pattern					
Relationship, fostered by illness’ narrative	(a) listen to the person	Patient: “But … if I don’t do anything? … What can happen to me?” Doctor: “The disease goes on … Listen … If you want, we can do something; we can also talk about it with your daughter” (RP. 9) Patient: “It’s been two years since we began the treatment; do I have to stop it?” Doctor: “Hmm … I understand perfectly; this treatment that I want to propose…” Patient: “Yes, I understand, you are offering me other treatments, but I’m tired.” (RP5)	Use of words and silence (the doctor left the room to listen to the person)	The tone of voice was modulated and adapted to what the doctor was communicating	Alternation of stillness and dynamic of postures, gestures, and facial expressions
	(b) attention to the person’s emotions	Patient: “Indeed, I am tired … very tired…” Doctor: “In fact, you are right! … We don’t really do it (that therapy), but now what we can offer you, if you want, is a therapy that contains the disease.” (RP 6) Patient: “And … if I don’t make it…?” Doctor: “Healing, you know, it’s not certain…. We speak of a cure, not healing, and you know it … we could search and have other opportunities for treatment.” (RP7)			
	(c) Using a narrative approach to describe the history of the person’s illness.	Doctor: “When you came for the blood test that we did, we found some cells that weren’t right …. Do you remember? We did the medullary needle biopsy because we needed to understand the situation properly—the most important thing is understanding that. So, Angela, the disease is confirmed.” (RP 6) Doctor: “When we saw each other, we made a plan…. It could be an important disease, but we said, because it happened before, that maybe we could keep the disease in check.” (RP 5) She doesn’t rush, uses a narrative approach, and shows proper patient and caregiver knowledge. (FN 10)			
Compassionate Sharing Pattern					
Sharing and reciprocity	(a) attention to the setting and control of emotions	Doctor says: “Time can be dedicated … in addition to the standard time that we use for a routine visit. We usually request the presence of a caregiver. And … [reflective pause] then, it’s expected that you know the person”. (Int. 7,2). “It is useful to prepare me well before communication, knowing the patient and caregivers in-dept.” (Int. 6,4) From nonverbal language, you can see that the doctor is listening to the patient; he/she keeps eye contact, he/she doesn’t seem bothered when stopped, but he/she starts listening again to his/her interlocutor. (ON.-RP 5)	Conscious use of intermixed words and silence (the doctor develops active listening and understanding)	The modulated tone of voice that adapts to the needs of understanding the other	Prevalence of dynamism and activity; involves leaning towards the person, a type of body language
	(b) using compassionate care	From the notes, it’s clear that the doctor is interested in the patient’s family situation and understanding the possible logistical problems involved in the treatment plan. (ON-RP 13)			
	(c) empathetic comprehension	From the notes, it’s clear that the doctor is leaving some pauses throughout the conversation; he/she is expanding the time to allow the patient to absorb the news and give him/her time to interrupt (ON-RP6) The doctors interviewed were very sensitive to these dimensions due to their deep understanding of what the patient was experiencing, including crying. (ON-Int 4) Doctor: “I try to give the patient enough time to express himself and to cry.” (Int. 4,8) Patient: “I’m really confused…” Doctor: “I know …. do you want to think about it for a while? I know …” Patient: “I’m afraid I won’t make it.” (RP7)			
	(d) sharing the care	The doctor and caregiver seem to agree on what to say to the patient. The interview ends with an agreement from both and a smile from the doctor. The closure appears to be relaxed. Both interlocutors managed to find a compromise. The doctor appears relieved at the result. (ON-RP 9)			

RP: role-playing; ON: observational note; Int: interview; FN bedside field notes.

## Data Availability

The data presented in this study are available on request from the corresponding author. The data are not publicly available due to ethical reasons.

## Supplementary Materials

The following supporting information can be downloaded at: https://www.mdpi.com/article/10.3390/ijerph19052585/s1, Supplementary File S1: SRQR checklist, Supplementary File S2: BAS scores.
