# Supplementary material for: Behavioral Patterns in Breaking Bad News Communication: An Ethnographic Study with Hematologists
Source: Int J Environ Res Public Health. 2022 Feb 23;19(5):2585. doi: 10.3390/ijerph19052585 (PMC8910064; doi:10.3390/ijerph19052585)
Supplement: Supplementary file 1 [file ijerph-19-02585-s001.zip › Supplementary File S2 - BAS scores.pdf]

International Journal of
Environmental Research and Public Health

MDPI

Supplemental Material S2. *BAS scores.*

Breaking bad news scale items	N° of bedside sessions with the item achieved/total bedside sessions		
	**Very/extremely well (4-5)**	**Quite well (3)**	**Slightly well, not at all well (1-2)**
In your opinion, how did the trainees conduct the encounters?			
Did the doctor carefully arrange the environment?	13/14	1/14	0
Did the doctor use an appropriate greeting and introduction?	14/14	0/14	0/14
Did the doctor show interest in the patient's current state of well-being and personal circumstances at the beginning of the interview?	9/14	1/14	4/14
Before breaking the news, did the doctor check what the patient knew already?	8/14	5/14	1/14
When breaking the news, did the doctor introduce it with sensitivity?	6/14	4/14	4/14
Did the doctor allow the patient to decide the level of detail and language used when delivering the news?	7/14	6/14	1/14
Did the doctor allow the patient to set the pace for the delivery of the news?	7/14	5/14	2/14
Did the doctor use an appropriate pause after giving the news?	6/14	5/14	3/14
Did the doctor specifically invite questions?	7/14	4/14	3/14
Did the doctor explicitly attempt to obtain a complete list of the patient's concerns?	5/14	4/14	5/14
Did the doctor explicitly check which areas were most important to the patient?	3/14	2/14	9/14
Information giving			
Did the doctor give information tailored to the patient's expressed concerns?	7/14	5/14	2/14
Did the doctor clearly explain any information given so that the patient understood?	11/14	1/14	2/14
Did the doctor manage to focus on any positive aspects?	14/14		
Was the content of the interview factually accurate?	8/14	6/14	0/14
General considerations			
How many concerns did the patient air?	5/14	6/14	3/14
How many of the key areas of the patient's concerns were touched upon?	4/14	5/14	5/14
Did patients note the psychosocial issues during the interview explored?	7/14	3/14	4/14
Did the doctor manage to appear supportive during the interview?	12/14	2/14	0/14
Did the doctor use appropriate body language during the interview?	12/14	2/14	0/14
Did the doctor avoid appearing clumsy during the interview?	11/14	3/14	0/14
Did the doctor tailor the pace of the interview to suit the patient?	11/14	3/14	0/14
Did the doctor manage the available time well?	9/14	4/14	1/14
